# Sesamolin serves as an MYH14 inhibitor to sensitize endometrial cancer to chemotherapy and endocrine therapy via suppressing MYH9/GSK3β/β-catenin signaling

**DOI:** 10.1186/s11658-024-00583-9

**Published:** 2024-05-02

**Authors:** Yibin Lin, Xiao Chen, Linping Lin, Benhua Xu, Xiaofeng Zhu, Xian Lin

**Affiliations:** 1 Department of Gynecology, Clinical Oncology School of Fujian Medical University, Fujian Cancer Hospital, Fuzhou, 350014 Fujian China; 2 Department of Intensive Care Unit, The First Affiliated Hospital, Fujian Medical University, Fuzhou, 350005 Fujian China; 3 Department of Intensive Care Unit, National Regional Medical Center, Binhai Campus of the First Affiliated Hospital, Fujian Medical University, Fuzhou, 350212 Fujian China; 4 Hunan Institute of Engineering, Xiangtan, 411100 Hunan China; 5 Department of Radiation Oncology, Fujian Medical University Union Hospital, Xinquan Road 29, Gulou District, Fuzhou, 350001 Fujian China; 6 Department of Oral Maxillo-Facial Surgery, The First Affiliated Hospital, Fujian Medical University, No. 20 Chazhong Road, Taijing District, Fuzhou, 350005 Fujian China; 7 Shenzhen Key Laboratory of Inflammatory and Immunology Diseases, No. 1120 Lianhua Road, Futian District, Shenzhen, 518036 Guangdong China; 8 Peking University Shenzhen Hospital, Shenzhen, 518036 Guangdong China; 9 Department of Oral Maxillo-Facial Surgery, National Regional Medical Center, Binhai Campus of the First Affiliated Hospital, Fujian Medical University, Fuzhou, 350212 China

**Keywords:** Endometrial cancer, MYH14, Sesamolin, Chemotherapy, Endocrine therapy

## Abstract

**Background:**

Endometrial cancer (EC) is one of the most common gynecological cancers. Herein, we aimed to define the role of specific myosin family members in EC because this protein family is involved in the progression of various cancers.

**Methods:**

Bioinformatics analyses were performed to reveal EC patients’ prognosis-associated genes in patients with EC. Furthermore, colony formation, immunofluorescence, cell counting kit 8, wound healing, and transwell assays as well as coimmunoprecipitation, cycloheximide chase, luciferase reporter, and cellular thermal shift assays were performed to functionally and mechanistically analyze human EC samples, cell lines, and a mouse model, respectively.

**Results:**

Machine learning techniques identified MYH14, a member of the myosin family, as the prognosis-associated gene in patients with EC. Furthermore, bioinformatics analyses based on public databases showed that MYH14 was associated with EC chemoresistance. Moreover, immunohistochemistry validated MYH14 upregulation in EC cases compared with that in normal controls and confirmed that MYH14 was an independent and unfavorable prognostic indicator of EC. MYH14 impaired cell sensitivity to carboplatin, paclitaxel, and progesterone, and increased cell proliferation and metastasis in EC. The mechanistic study showed that MYH14 interacted with MYH9 and impaired GSK3β-mediated β-catenin ubiquitination and degradation, thus facilitating the Wnt/β-catenin signaling pathway and epithelial–mesenchymal transition. Sesamolin, a natural compound extracted from *Sesamum indicum* (L.), directly targeted MYH14 and attenuated EC progression. Additionally, the compound disrupted the interplay between MYH14 and MYH9 and repressed MYH9-regulated Wnt/β-catenin signaling. The in vivo study further verified sesamolin as a therapeutic drug without side effects.

**Conclusions:**

Herein, we identified that EC prognosis-associated MYH14 was independently responsible for poor overall survival time of patients, and it augmented EC progression by activating Wnt/β-catenin signaling. Targeting MYH14 by sesamolin, a cytotoxicity-based approach, can be applied synergistically with chemotherapy and endocrine therapy to eventually mitigate EC development. This study emphasizes MYH14 as a potential target and sesamolin as a valuable natural drug for EC therapy.

**Graphical Abstract:**

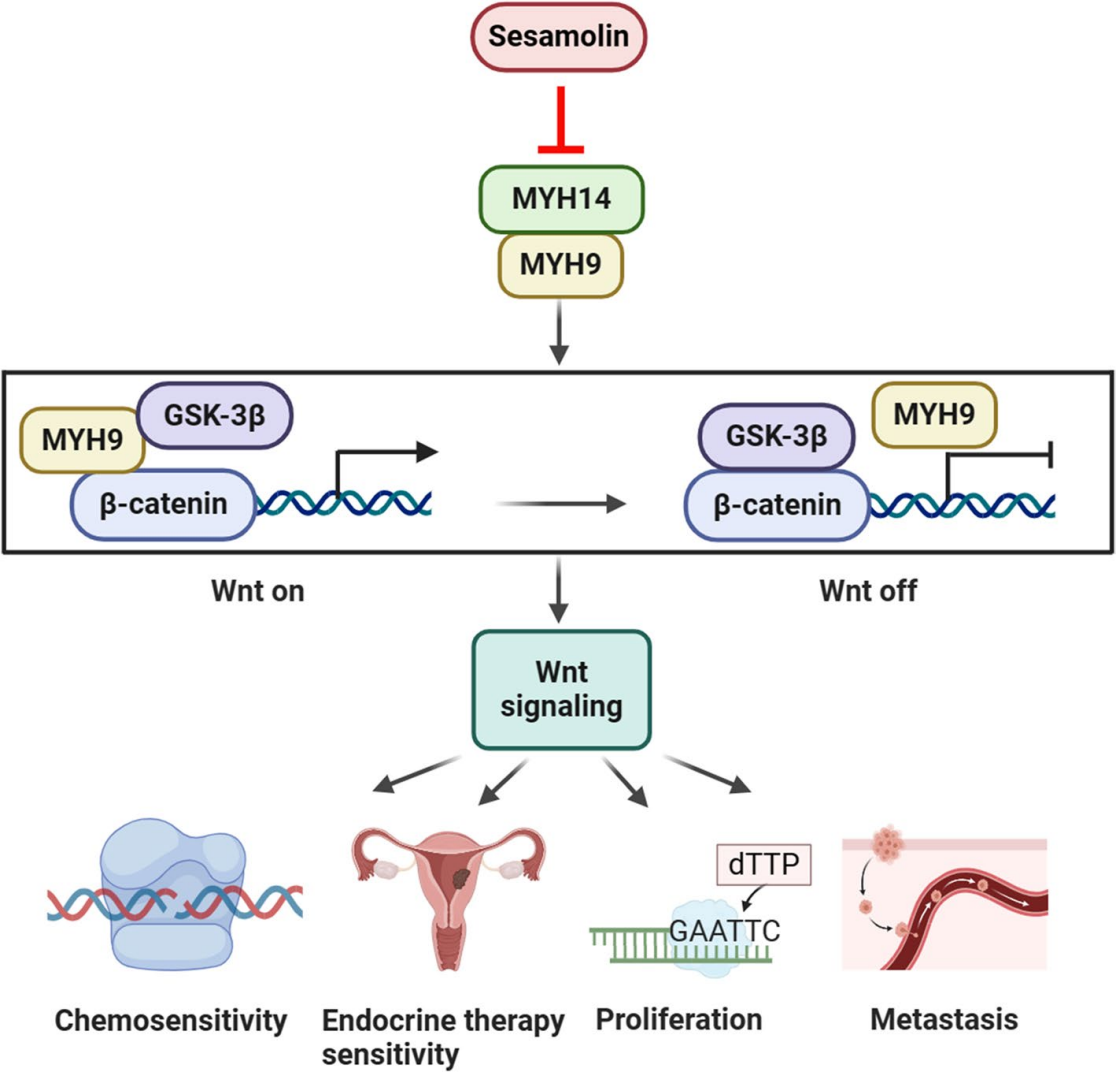

**Supplementary Information:**

The online version contains supplementary material available at 10.1186/s11658-024-00583-9.

## Background

Endometrial cancer (EC) is one of the most common gynecological cancers with increasing incidence [1, 2]. Despite the implementation of several available therapeutic strategies, the survival outcomes of patients with EC are unsatisfactory [3]. The combination of paclitaxel and carboplatin is the standard first-line chemotherapy for EC [4]. However, chemoresistance to this combination is one of the main factors contributing to poor EC prognosis [5]. Additionally, resistance to endocrine therapy is a worldwide issue in advanced and recurrent EC [6]. Thus, integrating new therapeutic targets may benefit the development of molecular-integrated adjuvant treatment, including targeted therapies [7]. Therefore, improving sensitivity to EC therapy may ameliorate poor patient prognosis associated with EC with the development of new targets or drugs.

The myosin family of molecular motors is ubiquitously expressed in eukaryotic organisms, and growing research shows the role of myosin family members in cancer progression [8]. Previously, we focused on nonmuscle myosin-IIs and found that nonmuscle myosin-IIA, namely MYH9, played an oncogenic role in hepatocellular [9] and nasopharyngeal carcinoma [10, 11]. MYH9 promoted cancer cell chemoresistance, proliferation, and metastasis, as well as it was associated with poor poor patient prognosis. MYO5B, a myosin family member, was responsible for poor patient outcomes and was involved in EC development [12]. Moreover, S100A4/NMII-related signaling was probably responsible for the development and maintenance of epithelial–mesenchymal transition (EMT)/stemness properties in uterine carcinosarcoma [13]. Nevertheless, the role of specific myosin family members in EC remains unclear.

Recently, machine learning has become a research hotspot. Increasing biological data and their inherent complexity have prompted machine learning applications in biology [14]. Many researchers follow machine learning approaches to elucidate potential biological mechanisms and construct predictive models. Machine learning techniques, such as random forest and lasso algorithms, can reveal prognosis-related genes in gynecological cancers [15, 16] and have been widely used in prognosticating gynecological malignancies [17].

Several natural bioactive compounds have been developed as novel cancer therapeutics. The anticancer effects of such bioactive phytochemicals are primarily manifested via two pathways, either by exerting cytotoxic effects on cancer cells without harming macromolecules such as DNA and enzymes in normal cells or by counteracting the oncogenic signaling axis that is activated in tumor cells [18]. Natural compounds can improve sensitivity to anticancer therapy [19]. Sesamolin, sesamin, and sesamol are the major natural lignans extracted from sesame seeds [20], and among them, sesamolin exhibits anticancer activities in several solid and hematologic tumors [21, 22]. However, its role in EC remains undetermined.

Herein, we aimed to identify a candidate myosin family member associated with prognosis in patients with EC using The Cancer Genome Atlas (TCGA) and Gene Expression Omnibus (GEO) databases by applying machine learning approaches. Additionally, the relationship between the identified myosin family member and sensitivity to EC therapy was investigated. Moreover, we searched for a drug candidate that directly targeted this myosin family member to improve patient prognosis and sensitivity to EC therapy. The effect of a natural compound, sesamolin, on EC progression mediated by an identified myosin family member, MYH14, was determined to assess the possible usage of MYH14 as a new target and sesamolin as a novel natural drug for EC therapy.

## Materials and methods

### Bioinformatics analysis of public data

The clinicopathological characteristics of patients with EC and RNA-sequencing fragments per kilobase of transcript per million mapped reads data of the uterine corpus endometrioid carcinoma (UCEC) dataset were downloaded from TCGA (https://portal.gdc.cancer.gov/). Nonexpressed genes were excluded from the analyses in the retrieved 541 patients with EC and 35 normal controls. The Gene Set Cancer Analysis (GSCA) database (http://bioinfo.life.hust.edu.cn/GSCA) was used to investigate an association between MYH14 expression and sensitivity to EC chemotherapy. RandomForest and glmnet packages in R were used to perform machine learning analyses to identify prognosis-associated myosins in patients with EC. The simple sample gene set enrichment analysis (ssGSEA), survival analysis, differential expression analyses, weighted gene coexpression network analysis (WGCNA), and correlation analyses were performed as described previously [23]. HALLMARK and Kyoto Encyclopedia of Genes and Genomes (KEGG) gene sets in the GSEA database (https://www.gsea-msigdb.org/gsea/index.jsp) were obtained for calculating signaling pathway activity. Platinum-drug-resistance-related genes in KEGG (https://www.kegg.jp/) were retrieved for gene set establishment as described previously [24]. Based on previous studies [24, 25], the GSVA package in R was used for ssGSEA to calculate a chemoresistance index and myosin activity. The WGCNA package in R was used to perform WGCNA in order to establish a gene coexpression network and reveal genes associated with EC. The oncoPredict package in R was used to evaluate chemoresistance in EC cases at a single sample level on the basis of gene expression data. Survival analyses were performed to define the best cut-off value by using the survminer package in R, and the survival curves of patients with EC were observed for low and high-expression groups.

### Immunohistochemistry (IHC)

Paraffin-embedded sections (4 μm) from 118 EC and 17 adjacent normal tissues were prepared for IHC. Patient consent and ethics approval from the local ethics committee were obtained (no. SQ2022-008-001). The sections were sequentially subjected to deparaffinization, rehydration, and antigen retrieval with citrate buffer. H_2_O_2_ (3%) and goat serum were used to eradicate endogenous peroxidase and block nonspecific antigens, respectively. Then, the sections were incubated with a primary antibody (Additional file 1: Table S1), followed by incubation with a secondary antibody. The resulting signals were detected by using a 3,3′-diaminobenzidine substrate, and the sections were stained with hematoxylin, sealed with neutral balsam, and analyzed under a microscope. Staining intensities were scored according to a previous study [26].

### Cell culture, transfection, and chemical compounds

Ishikawa (no: CL-0283, Procell) and KLE (no: CL-0133, Procell) cell lines with STR verification were cultivated in Dulbecco’s Modified Eagle Medium (DMEM)/F12 containing 10% fetal bovine serum (FBS) and routinely tested for mycoplasma contamination. MYH14 knockdown was performed with small interfering RNAs (siRNAs) designed and purchased from RiboBio Corporation (Guangzhou, China). MYH14 or MYH9 overexpression was achieved with plasmids purchased from Vigene Biosciences Corporation (Shandong, China). siRNAs and plasmids were used for cell transfection using Lipofectamine™ 3000 purchased from Invitrogen Corporation (Shanghai, China) according to the manufacturer’s instructions. After transfection, the EC cells were incubated for 48–72 h and collected for further analysis. Carboplatin (CAS: 41575-94-4), paclitaxel (CAS: 33069-62-4), and medroxyprogesterone acetate (CAS: 71-58-9) were obtained from TargetMol Biotechnology Corporation (Boston, USA). Sesamolin (CAS: 526-07-8) was purchased from Herbest Biotechnology Corporation.

### Quantitative reverse transcription polymerase chain reaction (qRT–PCR)

Total RNA isolation was performed with harvested EC cells. Then, reverse transcription was performed to obtain complementary DNA that was used as a template. qPCR was performed using Roche Lightcycler 480II with qPCR reagents purchased from Accurate Biotechnology Corporation (Changsha, China) and specific primers (Additional file 1: Table S2). The relative RNA levels were determined by the 2^−ΔΔCt^ method.

### Western blot analysis

EC cells were collected for cellular protein extraction. A BCA protein assay kit purchased from Beyotime Corporation (Shanghai, China) was used to quantify the proteins. After denaturation, the proteins were separated by sodium dodecyl sulfate polyacrylamide gel electrophoresis, transferred to a polyvinylidene fluoride membrane, and incubated with primary antibodies (Additional file 1: Table S1). Then, the proteins were incubated with secondary antibodies. The resulting chemiluminescent signal was measured to indicate protein levels, and Bio-Rad ChemiDocTM XRS+ (Bio-Rad, USA) was used to capture images.

### Colony formation assay

The treated EC cells and controls (1000 cells/well) were cultivated in six-well plates. After 14 days of cultivation, the generated colonies were fixed with methanol for 20 min, stained with 2.5% crystal violet for 40 min, and subjected to image recording. The number of colonies was calculated for further analysis.

### Cell counting kit 8 (CCK-8) assay

A CCK-8 reagent purchased from APExBIO (Huston, USA) was used to detect cell viability in 96-well plates. The treated EC cells and controls were incubated with the CCK-8 reagent for 2 h at 37 °C. After incubation, their absorbance values were measured using a microplate reader (BioTek, USA) at 450 nm.

### Immunofluorescence

EC cells (5000 cells/well) were seeded in 96-well plates for cell adhesion. The treated cells and controls were fixed with 4% paraformaldehyde, permeabilized with 0.5% Triton X-100, and incubated with a primary antibody (Additional file 1: Table S1). An Alexa Fluor® 488-conjugated secondary antibody and 4′,6-diamidino-2-phenylindole were used for co-staining the EC cells. A fluorescence microscope was used to capture images.

### Wound healing assay

EC cells plated in 12-well plates were allowed to grow into a confluent monolayer. Subsequently, the cells were scratched using a pipette tip. After wounding at 0, 24, and 48 h, the cells were fixed and stained with 2.5% crystal violet. Wound healing was recorded under a microscope.

### Transwell assay

Transwell assay was performed as described previously [27]. Briefly, precoated and uncoated transwells were used for invasion and migration assays, respectively. A total of 1.0 × 10^5^ EC cells in FBS-free DMEM/F12 were plated into the upper chamber of the transwell system, followed by filling the lower chamber with DMEM/F12 containing 10% FBS. The migrated and invaded cells were fixed with methanol for 20 min and stained with 2.5% crystal violet for 40 min at 24 h and 48 h, respectively. A microscope was used to record images.

### Coimmunoprecipitation (Co-IP)

The Pierce Co-IP kit (No. 26149) purchased from Thermo Scientific Corporation (Shanghai, China) was used to perform the Co-IP assay according to the manufacturer’s instructions. Briefly, antibodies were covalently coupled onto an amine-reactive resin to avoid the co-elution of the heavy and light chains of the antibodies. They were immobilized to amine-reactive resin and subjected to incubation. Then, proteins were extracted from the EC cells by ice-cold IP lysis/wash buffer and quantified. A total of 5 mg of the proteins were pre-incubated using the control agarose resin to eliminate nonspecific binding, followed by incubation with antibodies (Additional file 1: Table S1) or a negative control overnight at 4 °C on a rotator. Agarose-coupled antibodies were used for immunoprecipitation, and the bound proteins were eluted for silver staining, mass spectrometry, or western blotting.

### Cycloheximide (CHX) chase assay

As described previously [28], a CHX chase assay was performed to measure the protein half-time. EC cells were incubated with CHX (50 µg/mL) at the indicated time. Thereafter, the proteins were harvested from EC cells using a lysis buffer and quantified using a BCA protein assay kit. The obtained proteins were analyzed by western blotting to measure their half-life.

### Luciferase reporter assay

Wnt signaling activity was detected using the luciferase assay system as described previously [28]. Briefly, EC cells were co-transfected with TOPFlash or FOPFlash with pRL reporter plasmids purchased from Millipore (Billerica, MA, USA). After transfection and culturing for 48 h, Dual-Luciferase Reporter Assay System (Promega Corporation) with a BioTek luminometer was used to measure luciferase activity. Firefly and Renilla luciferase activities were detected by the luminometer and normalized.

### Molecular docking

Molecular docking was performed using Discovery Studio. The SDF file of sesamolin or sesamol and the structure of the MYH14 protein (5JLH) were downloaded from the PubChem and PDB databases, respectively. Discovery Studio was used for ligand removal, hydrogen addition, water removal, and amino acid optimization and repair. A CDOCKER docking module in Discovery Studio was adopted for docking calculation and analysis.

### Cellular thermal shift assay (CETSA)

CETSAs were performed as described previously [29]. Briefly, EC cells were incubated with sesamolin or a solvent (control) and collected for protein extraction. Cell lysates were prepared by a freeze–thaw method using liquid nitrogen and a heating block set at 25 °C. The soluble fraction was diluted and divided into eight aliquots for 3 min of heating at 40 °C, 45 °C, 50 °C, 55 °C, 60 °C, 65 °C, 70 °C, and 75 °C followed by 3 min of cooling at room temperature. The soluble fraction separated by centrifugation was harvested for western blotting.

### In vivo study

The Institutional Animal Care and Use Committee of the hospital authorized the experimental protocols (no. 2021-762). According to relevant regulatory standards, a specific pathogen-free environment was maintained for raising female BALB/c nude mice. Mice aged 4–5 weeks obtained from Shanghai SLAC Experimental Animal Corporation were subcutaneously inoculated with 2.0 × 10^6^ Ishikawa cells per mouse. The mice were randomized into four groups (*n* = 5). The combination of carboplatin (16 mg/kg) and paclitaxel (20 mg/kg) was intraperitoneally injected as described previously [30]. Sesamolin was orally administered at 30 mg/kg and 60 mg/kg. At the end of the observation, the mice were sacrificed with euthanasia, and xenografts were preserved for subsequent analyses.

### Statistical analysis

SPSS 22.0 and R software were used for statistical analyses. Normal distribution variables were presented as the mean ± standard deviation from at least three independent assays. Nonnormal distribution variables were calculated as the median ± range. The Wilcoxon rank-sum test and the Student’s *t*-test were used to estimate the statistical difference between the two groups. The one-way ANOVA was performed to assess statistical significance among multiple groups. The correlation analyses were performed using Spearman’s rank correlation test. The log-rank test was used to measure a survival difference and Kaplan–Meier survival curves were plotted. Cox regression models were used to determine the prognostic values of factors related to the survival of patients with EC. *P* < 0.05 was considered indicative of statistical significance.

## Results

### Machine learning techniques combined with WGCNA identified MYH14 as a prognosis-associated gene in patients with EC

**Fig. 1:**
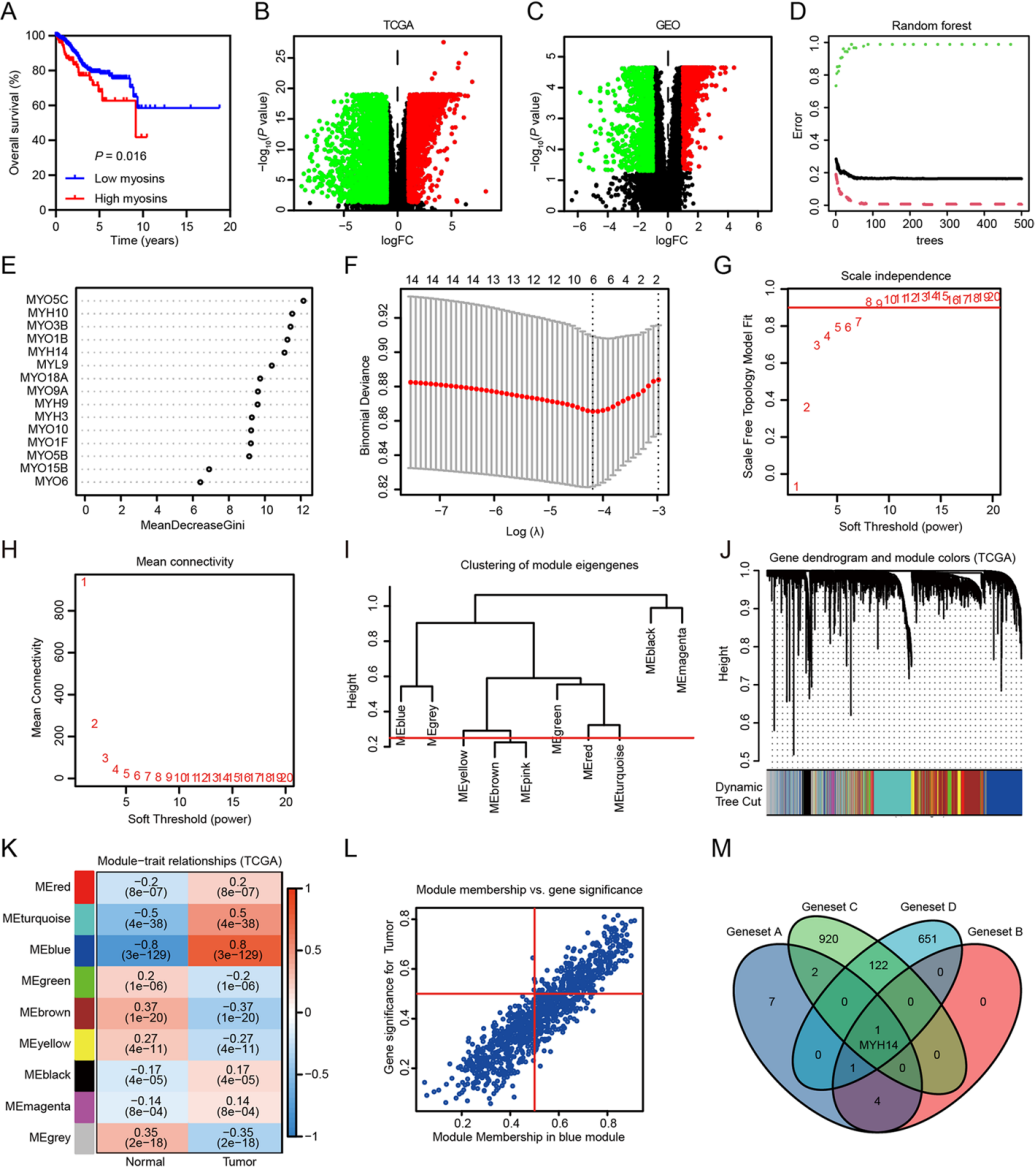
Bioinformatics analyses of the public database identify the prominent myosin family members correlated with EC prognosis. **A** Survival analysis displaying the relationship between myosin activities calculated by ssGSEA and the overall survival of EC patients in the TCGA UCEC dataset. Kaplan–Meier curves were plotted using the log-rank test. **B**, **C** Volcano plots presenting differentially expressed genes between EC and normal controls in TCGA UCEC and GSE17025 datasets, respectively. **D**, **E** The random forest model was applied to rank the identified 15 myosins that correlated with EC patient outcomes. The relationship between number of decision trees and model error; *x* axis, number of decision trees; and *y* axis, error rate of the obtained model are shown. Importance of all variables in the random forest classifier as determined by the Gini coefficient method; *x* axis, mean decrease in Gini index; and *y* axis, variables are shown. **F** The lasso regression model was used to identify prognosis-associated myosins in EC patients. Tenfold cross-validation was used to tune the parameters in the lasso model. **G**, **H** Scale independence and mean connectivity were plotted to reveal the soft threshold and scale-free topology model fit index according to the TCGA UCEC dataset. **I**, **J** A GeneTree and a cluster dendrogram were established based on the soft threshold according to the TCGA UCEC dataset. Different colors represent different co-expression modules. **K** The heatmap showing the relationship between gene modules and EC according to the TCGA UCEC dataset. Each row represents a module, and each column represents a clinical status. **L** The scatter plots presenting the association between genes and EC in the MEblue module. **M** A Venn diagram showing the overlapping gene correlated with the prognosis of EC patients. *EC* Endometrial cancer, *GEO* Gene Expression Omnibus, *ssGSEA* simple sample gene set enrichment analysis, *TCGA* The Cancer Genome Atlas, *UCEC* Uterine corpus endometrioid carcinoma

To investigate the role of the myosin family in EC, a total of 52 myosin family members were considered for myosin activity calculation by the ssGSEA method (Additional file 1: Table S3). This activity was calculated, and the survival analyses suggested that high myosin activity indicated poor patient prognosis in EC according to TCGA (Fig. 1A). After confirming myosin involvement in EC progression, we investigated the most significant myosin associated with EC. First, a differential analysis was performed and 5494 differentially expressed genes were recognized between the EC samples and the normal controls according to TCGA UCEC and GSE17025 datasets (Fig. 1B, C). The differentially expressed gene included 15 myosins, and a random forest model was used to rank the identified 15 myosins that were correlated with patient outcomes (Fig. 1D, E) (geneset A). Moreover, the lasso regression model validated MYH10, MYH14, MYO1F, MYO3B, MYO5C, and MYO9A as prognosis-associated myosins in patients with EC (Fig. 1F) (geneset B). Furthermore, WGCNA was performed with the 5494 differentially expressed genes, and 1045 genes in the MEblue module (geneset C) as well as 775 genes in the Mebrown module (geneset D) were identified as genes closely correlated with EC according to TCGA UCEC (Fig. 1G–L) and GSE17025 datasets (Additional file 1: Fig. S1A–F), respectively. The intersection of the four gene sets showed MYH14 as the overlapping gene (Fig. 1M), indicating MYH14 as a prognosis-associated gene in patients with EC.

**Fig. 2:**
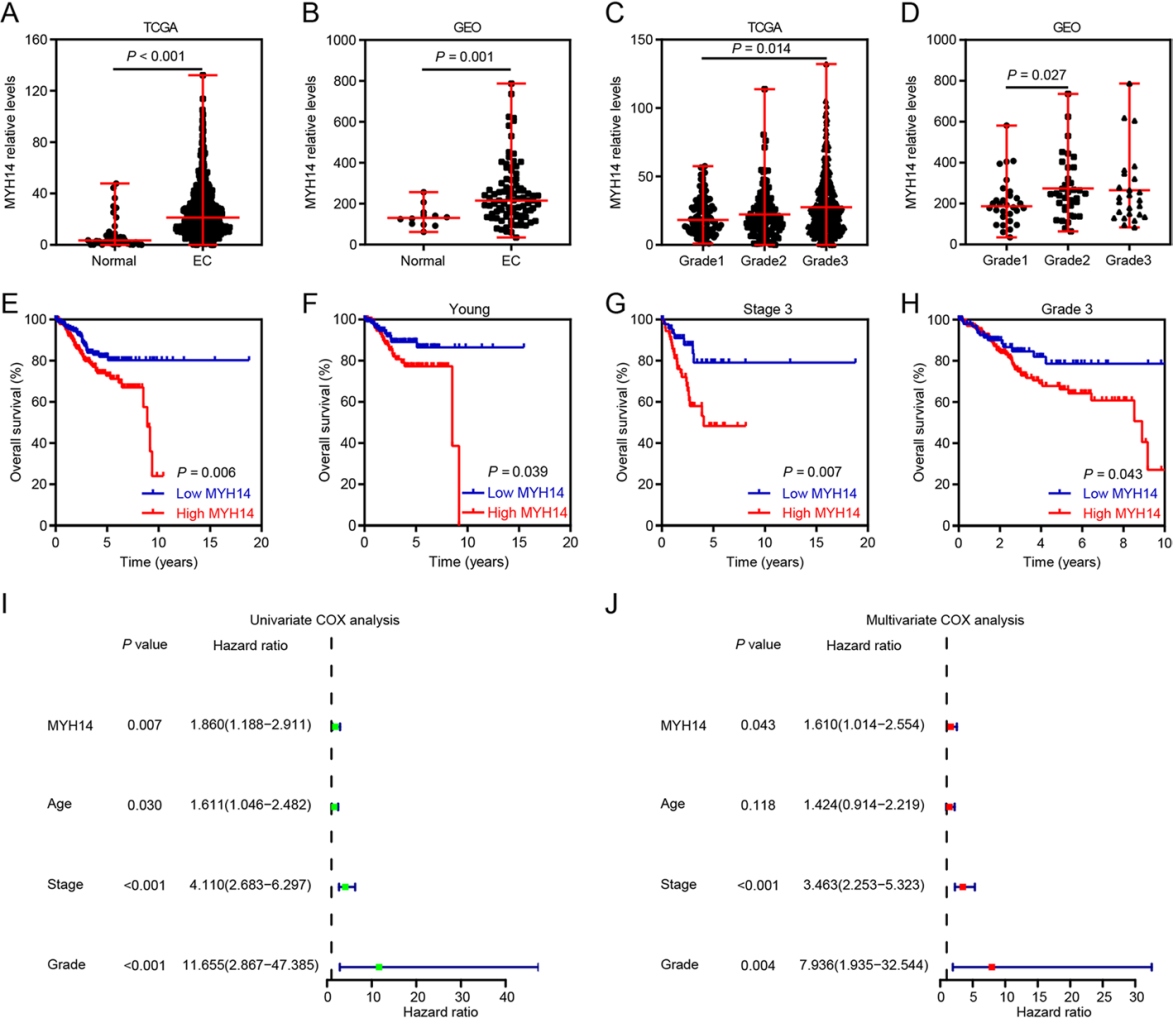
MYH14 expression was elevated in EC and conferred poor overall survival to patients according to the public database. **A**, **B** MYH14 expression was differentially expressed in EC and normal controls according to the TCGA UCEC and GSE17025 datasets. **C**, **D** MYH14 expression was differentially expressed in grades 1 and 2, and three EC tissues according to the TCGA UCEC and GSE17025 datasets. **E** Survival analysis displaying the association between MYH14 levels and the overall survival of EC patients. **F**–**H** Subgroup analyses exhibiting the overall survival time of EC patients in young (age ≤ 63 years) (**F**), stage 3 (**G**), and grade 3 (**H**). Kaplan–Meier curves were plotted using the log-rank test. **I**, **J** Univariate and multivariate Cox analyses disclosing the association between prognosis, clinicopathologic features, and MYH14 levels in EC patients. *EC* endometrial cancer, *GEO* Gene Expression Omnibus, *TCGA* The Cancer Genome Atlas

The role of MYH14 in EC was further investigated by performing bioinformatics analyses. In both TCGA UCEC and GSE17025 datasets, the differential analyses indicated MYH14 upregulation in EC compared with that in normal controls (Fig. 2A, B). Furthermore, MYH14 expression was upregulated in high-grade EC samples compared with that in low-grade EC samples (Fig. 2C, D). The survival analyses based on the best cut-off value revealed a positive correlation between poor overall survival of patients with EC and upregulated MYH14 expression (Fig. 2E). Additionally, the subgroup analyses according to the best cut-off value showed that upregulated MYH14 expression conferred poor prognosis of patients with EC with young age, stage 3, and grade 3 (Fig. 2F–H). Then, univariate and multivariate Cox hazard analyses were performed to verify the relationship between MYH14 expression and patient prognosis in EC. The univariate Cox analysis indicated that upregulated MYH14 expression, elder age, advanced EC stage, and high grade conferred the patients with poor overall survival (Fig. 2I). The multivariate Cox analysis indicated that MYH14 expression independently served as a predictor of the overall survival time of patients with EC (hazard ratio: 1.610, 95% confidence interval: 1.014–2.554, *P* = 0.043) (Fig. 2J).

Collectively, these findings indicated that MYH14 functioned as a possible diagnostic and prognostic predictor of EC.

### MYH14 levels were potentially associated with EC chemoresistance

Based on the association between MYH14 expression and the prognosis of patients with EC, ssGSEA was performed to determine the activities of biological processes and signaling pathways and correlate them with MYH14 expression. The correlation analyses revealed the positive association between TGF-β signaling, Wnt/β-catenin signaling, EMT, PI3K/AKT/mTOR signaling, Notch signaling, and MYH14 expression based on the HALLMARK geneset. Notably, the correlation analyses also revealed the positive association between TGF-β signaling, Wnt signaling, phosphatidylinositol signaling system, mTOR signaling, Notch signaling, and MYH14 expression based on the KEGG dataset (Additional file 1: Fig. S2A, B).

We further investigated the relationship between MYH14 expression and chemoresistance in patients with EC. Using ssGSEA, we calculated the platinum drug resistance index for patients with EC based on the TCGA database as we previously reported [24]. The analysis revealed a positive association between MYH14 expression and the platinum drug resistance index in the TCGA database on patients with EC (Additional file 1: Fig. S3A). Moreover, we used the oncoPredict tool in R and found a positive correlation between MYH14 expression and paclitaxel drug resistance in the GSE17025 dataset (Additional file 1: Fig. S3B). Furthermore, the GSCA database revealed that MYH14 levels were associated with cisplatin (correlation = 0.189, *P* < 0.001) and paclitaxel (correlation = 0.169, *P* < 0.001) drug resistance.

Altogether, these results suggested that MYH14 might be a chemoresistance-related oncogene in EC.

### MYH14 expression was associated with EC pathology

**Fig. 3:**
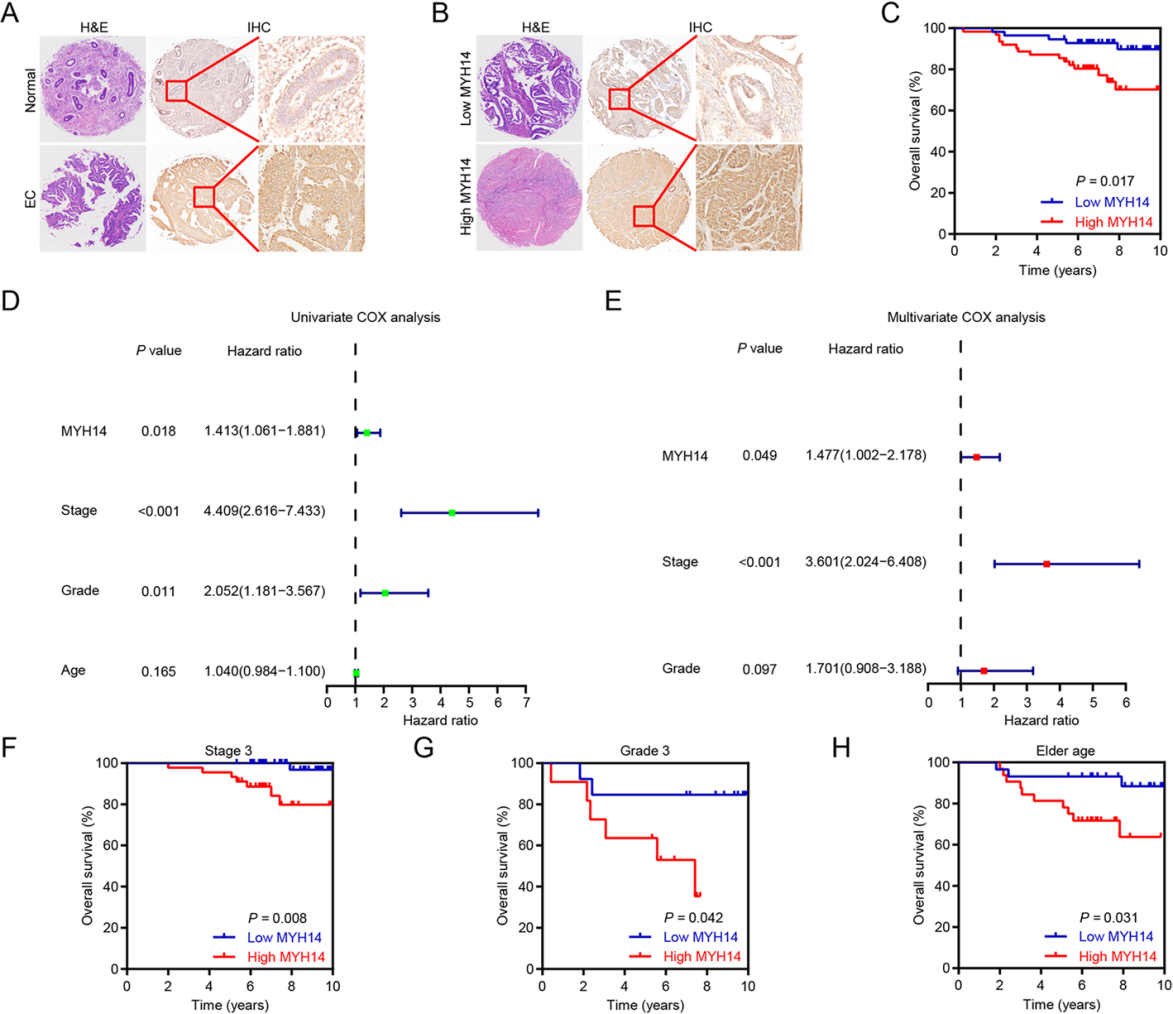
MYH14 levels detected by IHC were elevated in EC and associated with poor patient prognosis. **A** Representative images presenting the differential MYH14 expression between EC and normal controls. **B** Representative images presenting differential MYH14 expression in EC. **C** Survival analysis revealing the association between MYH14 levels and the overall survival of EC patients. **D**, **E** Univariate and multivariate Cox analyses disclosing the association between MYH14 expression, clinicopathologic features and prognosis of EC patients. **F**–**H** Subgroup analyses were performed to elucidate the overall survival time of EC patients in stage 3 (**F**), grade 3 (**G**), and elderly (age > 55 years) (**H**). Kaplan–Meier curves were plotted using the log-rank test. *EC* endometrial cancer, *H&E* hematoxylin and eosin, *IHC* immunohistochemistry

To further determine the clinical significance of MYH14 in EC, we performed an IHC analysis using 118 EC samples and 17 adjacent normal controls. The results revealed increased MYH14 expression in EC than in adjacent normal controls (Fig. 3A). Additionally, MYH14 expression varied among EC tissues (Fig. 3B). Further analysis indicated a positive correlation between MYH14 protein levels and Ki67 expression (Additional file 1: Table S4). Survival analysis showed a positive association between MYH14 expression and poor overall survival in patients with EC (Fig. 3C). Univariate and multivariate Cox hazard analyses confirmed MYH14 expression as an unfavorable and independent prognostic indicator for overall survival in patients with EC (hazard ratio: 1.477, 95% confidence interval: 1.002–2.178, *P* = 0.049) (Fig. 3D, E). Stratified analyses based on stage, grade, or age showed that MYH14 expression remained positively associated with poor overall survival in patients with EC (Fig. 3F–H).

To summarize, these findings revealed the crucial role of MYH14 in the progression of EC and suggested its potential clinical value as a biomarker.

### MYH14 knockdown ameliorated therapy sensitivity and inhibited cell proliferation and metastasis in EC

**Fig. 4:**
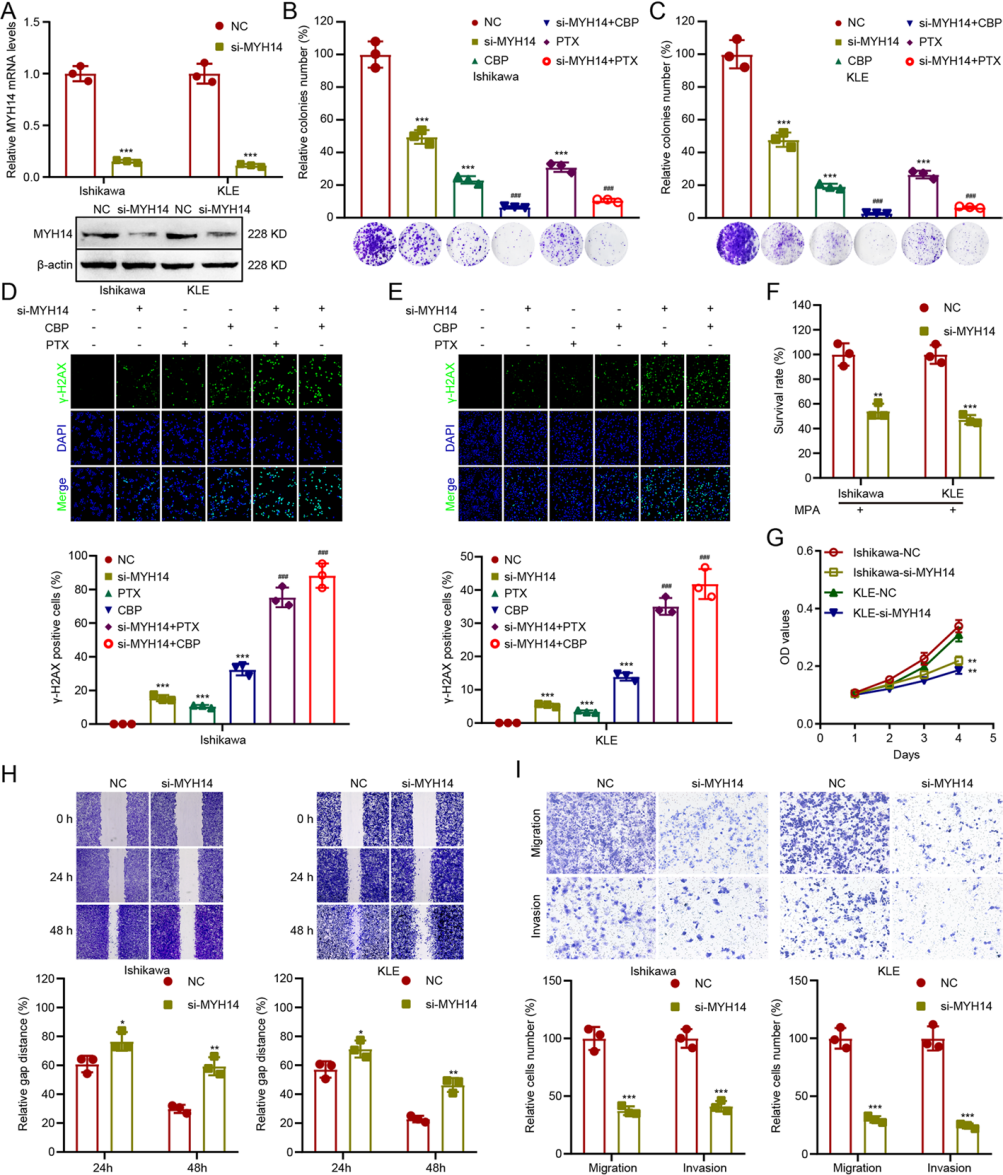
MYH14 knockdown improved therapeutic sensitivity and suppressed cell proliferation and metastasis in EC. Cells were treated with CBP, PTX, or MPA for 48 h and subjected to further experiments. **A** qPCR and western blot assays were applied to assess MYH14 levels in MYH14-silenced Ishikawa and KLE cells and the controls. **B**–**E** Colony formation (**B**, **C**) and immunofluorescence (**D**,** E**) assays were adopted to measure the effect of MYH14 on the chemosensitivity of Ishikawa and KLE cells. **F** CCK-8 assays were employed to measure the sensitivity to endocrine therapy of MYH14-silenced Ishikawa and KLE cells and the controls. Ishikawa and KLE cells were treated with 40 μM and 50 μM MPA, respectively. **G** CCK-8 assays were used to determine the proliferation of MYH14-silenced Ishikawa and KLE cells and the controls. **H**, **I** Wound healing (**H**) and transwell (**I**) assays were applied for evaluating the metastasis of MYH14-silenced Ishikawa and KLE cells and the controls. * *P* < 0.05, ** *P* < 0.01, and *** *P* < 0.001 versus the control group. ^###^ *P* < 0.001 versus the CBP or PTX group. *EC* endometrial cancer, *CBP* carboplatin, *MPA* medroxyprogesterone acetate, *PTX* paclitaxel, *qPCR* quantitative RT–PCR

The biological functions of MYH14 in the oncogenic process and signaling pathways were explored on the basis of the results obtained from bioinformatics analyses. MYH14 expression was silenced using siRNA in the Ishikawa and KLE cells (Fig. 4A). Subsequently, we evaluated the effect of carboplatin and paclitaxel on EC cells to determine the appropriate concentration for subsequent experiments (Additional file 1: Fig. S4A–D). Colony formation assays revealed that MYH14 knockdown sensitized EC cells to carboplatin and paclitaxel (Fig. 4B, C). Immunofluorescence analysis of the DNA damage marker (γH2AX) reported that MYH14 depletion increased the number of γ-H2AX-positive cells in carboplatin and paclitaxel-treated Ishikawa and KLE cells (Fig. 4D, E), further confirming the role of MYH14 in regulating EC chemosensitivity. Intriguingly, silencing MYH14 also sensitized EC cells to progesterone (Fig. 4F). Furthermore, MYH14 depletion was associated with a reduction in EC cell proliferation, as evidenced by CCK-8 assays (Fig. 4G). Wound healing and transwell assays further revealed the inhibitory effect of MYH14 silencing on the migration and invasion of EC cells (Fig. 4H, I).

These results indicated the inhibitory role of MYH14 in EC chemosensitivity and endocrine therapy sensitivity and suggested the stimulative role of MYH14 in the proliferation and metastasis of EC.

### MYH14 interacted with MYH9 to facilitate Wnt/β-catenin signaling

**Fig. 5:**
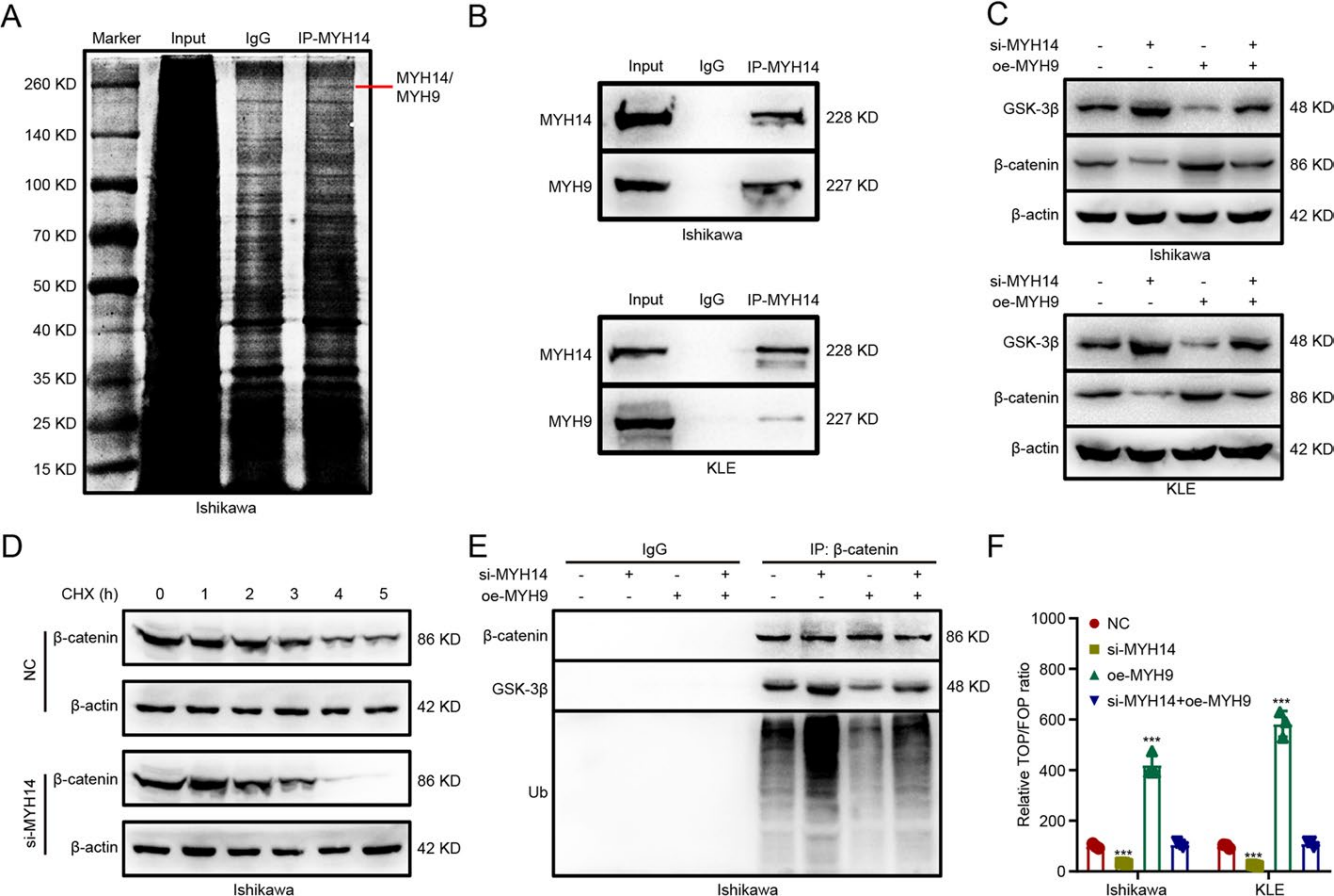
MYH14 interacted with MYH9 to facilitate Wnt/β-catenin signaling. **A** Co-IP combined with silver staining identifying the interacting protein of MYH14. **B** Co-IP assays detecting the interplay between MYH14 and MYH9 in Ishikawa and KLE cells. **C** Western blot assays showing the effect of MYH14 and MYH9 on GSK3β and β-catenin expression. **D** CHX chase assay showing the effect of MYH14 on the half-life of β-catenin protein. **E** Co-IP assays were conducted for measuring the effect of MYH14 and MYH9 on the formation of GSK3β/β-catenin/ubiquitin complex in Ishikawa cells treated with MG132. **F** TOP/FOP luciferase reporter assays were applied for detecting the effect of MYH14 and MYH9 on Wnt signaling activity. *** *P* < 0.001 versus the control group. *CHX* cycloheximide, *Co-IP* coimmunoprecipitation

To elucidate the potential mechanism of MYH14 in promoting EC progression, we investigated the interacting protein of MYH14. Co-IP along with mass spectrometry identified MYH9 as a potential interacting protein of MYH14 (Fig. 5A). Our previous studies have shown that MYH9 attenuated the GSK3β-mediated ubiquitination and degradation of β-catenin, thereby facilitating Wnt signaling activity and promoting proliferation, metastasis, and chemoresistance of cancer cells [9]. Additionally, the aforementioned bioinformatics analyses indicated the involvement of MYH14 in Wnt signaling. Therefore, we performed further investigations to determine the relationship between MYH14 and MYH9. Co-IP confirmed the binding between MYH14 and MYH9 in EC cells using Co-IP (Fig. 5B). The subsequent experiments aimed to determine how MYH14 affected the MYH9-mediated ubiquitination and degradation of β-catenin. MYH14 knockdown resulted in the upregulation of GSK3β expression and the downregulation of β-catenin expression, which could be reversed by MYH9 overexpression (Fig. 5C). Additionally, CHX chase assay showed that MYH14 depletion shortened the half-life of β-catenin protein (Fig. 5D). Furthermore, silencing MYH14 facilitated the formation of the GSK3β/β-catenin/ubiquitin complex, which could be reversed by MYH9 overexpression (Fig. 5E). Moreover, MYH14 downregulation restrained the activation of Wnt signaling, and this impact could be recovered by MYH9 overexpression (Fig. 5F). These results collectively indicated that MYH14 attenuated the GSK3β-mediated ubiquitination and degradation of β-catenin through its interaction with MYH9, thereby activating Wnt signaling in EC.

### Sesamolin directly targeted MYH14 to attenuate EC progression

**Fig. 6:**
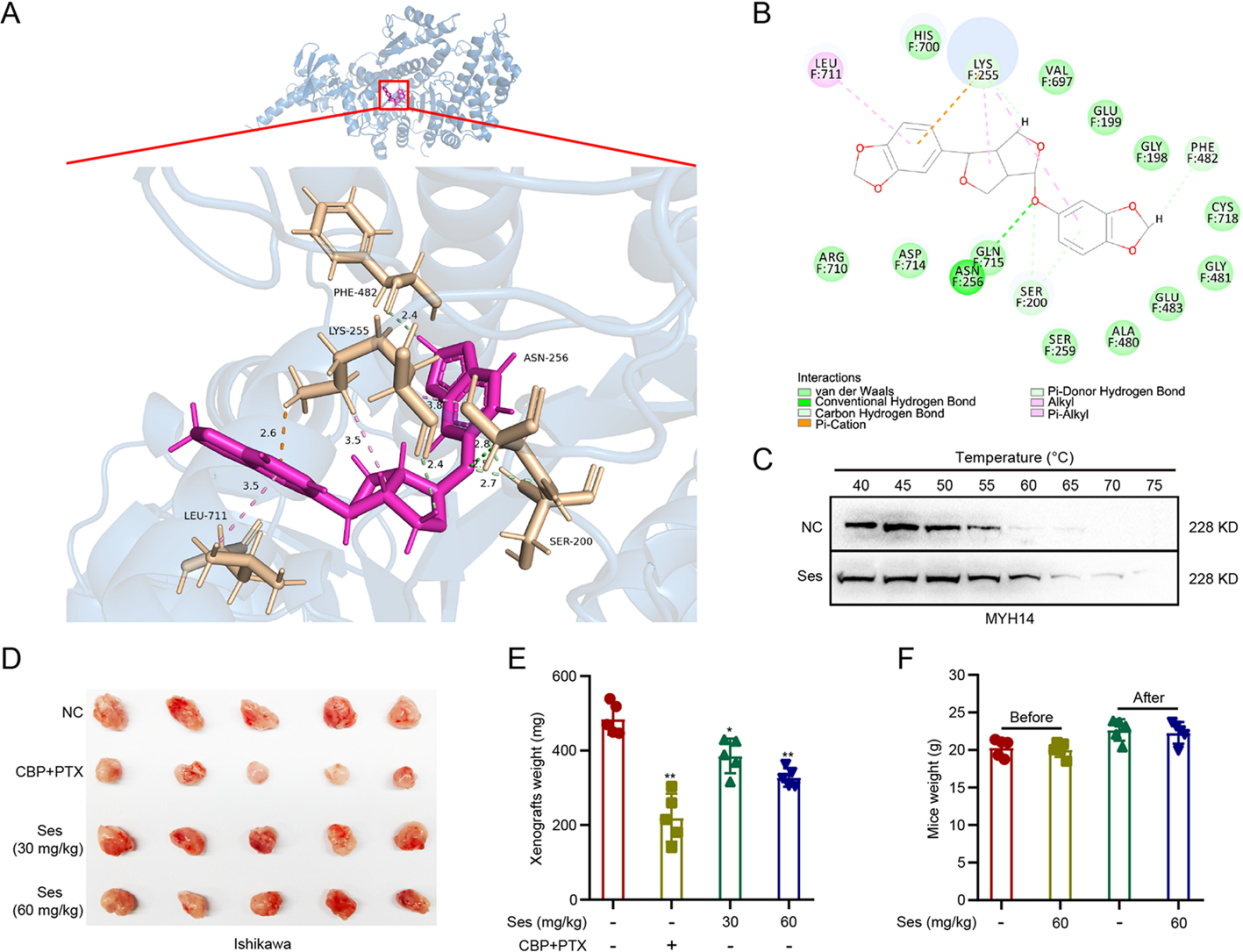
Sesamolin directly targeted MYH14 to attenuate EC progression. **A**, **B** Molecular docking revealing the potential interaction between sesamolin and MYH14. Three- and two-dimensional pattern diagrams showing the binding between sesamolin and MYH14. **C** CETSA was performed to assess the effect of sesamolin on the protein stability of MYH14. Cells were treated with Ses for 48 h. **D**, **E** An in vivo study was conducted to show the effect of sesamolin on the size and weight of xenografts. **F** For in vivo toxicity assessment, the body weight of mice was measured before and after sesamolin administration. * *P* < 0.05, and ** *P* < 0.01 versus the control group. *EC* endometrial cancer, *CBP* carboplatin, *CETSA* cellular thermal shift assay, *PTX* paclitaxel, *Ses* sesamolin

**Fig. 7:**
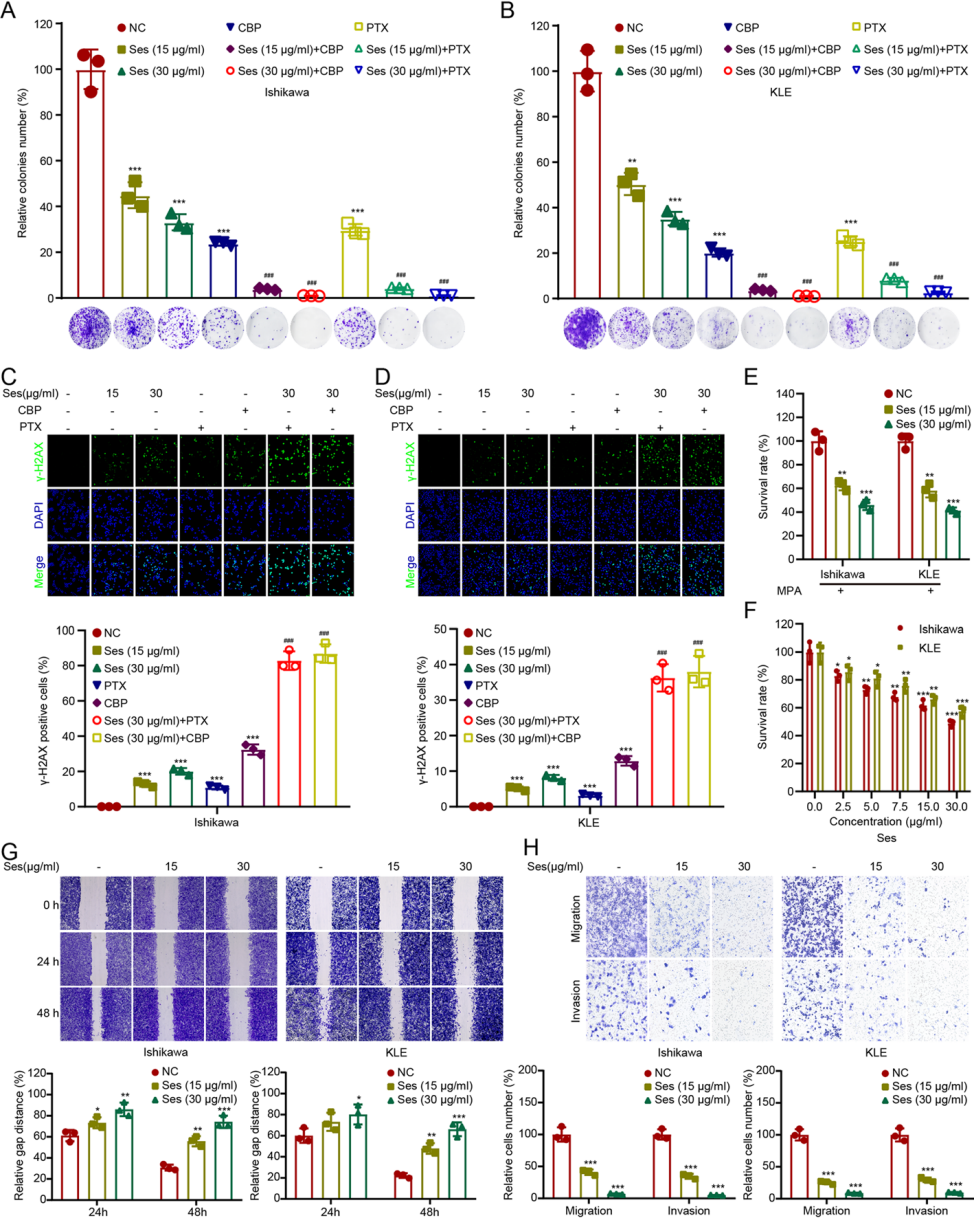
Sesamolin improved therapeutic sensitivity and attenuated cell proliferation and metastasis in EC. Cells were treated with CBP, PTX, MPA, and/or Ses for 48 h and subjected to further experiments. **A**–**D** Colony formation (**A**, **B**) and immunofluorescence (**C**, **D**) assays were adopted for evaluating the effect of sesamolin on the chemosensitivity of Ishikawa and KLE cells. **E** CCK-8 assays were adopted to measure the endocrine therapy sensitivity of sesamolin-treated Ishikawa and KLE cells and the controls. Ishikawa and KLE cells were treated with 40 μM and 50 μM MPA, respectively. **F** CCK-8 assays were conducted to reveal the proliferation of sesamolin-treated Ishikawa and KLE cells and the controls. **G**,** H** Wound healing (**G**) and transwell (**H**) assays were applied to elucidate the metastasis of sesamolin-treated Ishikawa and KLE cells and the controls. * *P* < 0.05, ** *P* < 0.01, and *** *P* < 0.001 versus the control group. ^###^ *P* < 0.001 versus the CBP or PTX group. *EC* endometrial cancer, *CBP* carboplatin, *MPA* medroxyprogesterone acetate, *PTX* paclitaxel, *Ses* sesamolin

After establishing the oncogenic role of MYH14 in EC, we searched for a potential drug targeting MYH14. Previous research has suggested a direct interaction between sesamol and MYH14 [31]. Because sesamolin was shown to have superior anti-cancer properties than its metabolite sesamol [32], we investigated whether sesamolin or its metabolite sesamol could target MYH14. Molecular docking showed a higher probability of interaction between sesamolin and MYH14 compared with sesamol, as determined by CDOCKER interaction energy (Fig. 6A, B). Subsequently, we determined the potential interaction between sesamolin and MYH14. CETSA showed that sesamolin increased the protein stability of MYH14, confirming MYH14 as a direct target of sesamolin (Fig. 6C). An in vivo study confirmed the suppressive role of sesamolin on EC progression, as indicated by the evaluation of the size and weight of xenografts (Fig. 6D, E). Notably, there was no significant difference in the body weight of mice between the sesamolin-treated group and the control group, suggesting no distinct toxicity of sesamolin in vivo (Fig. 6F). Then, the biological function of sesamolin in EC progression was further determined. Colony formation assays showed that sesamolin sensitized EC cells toward carboplatin and paclitaxel in a dose-dependent manner (Fig. 7A, B). Immunofluorescence analysis indicated that sesamolin increased the percentage of γ-H2AX-positive cells in carboplatin and paclitaxel-treated EC cells (Fig. 7C, D). Additionally, sesamolin restored sensitivity to progesterone treatment in a dose-dependent manner, and it reduced the proliferation, migration, and invasion of EC cells (Fig. 7E–H). These findings suggested that sesamolin acted as an MYH14 inhibitor, inhibiting EC progression by directly targeting MYH14.

### Sesamolin disrupted the interplay between MYH14 and MYH9 to inactivate Wnt/β-catenin signaling

**Fig. 8:**
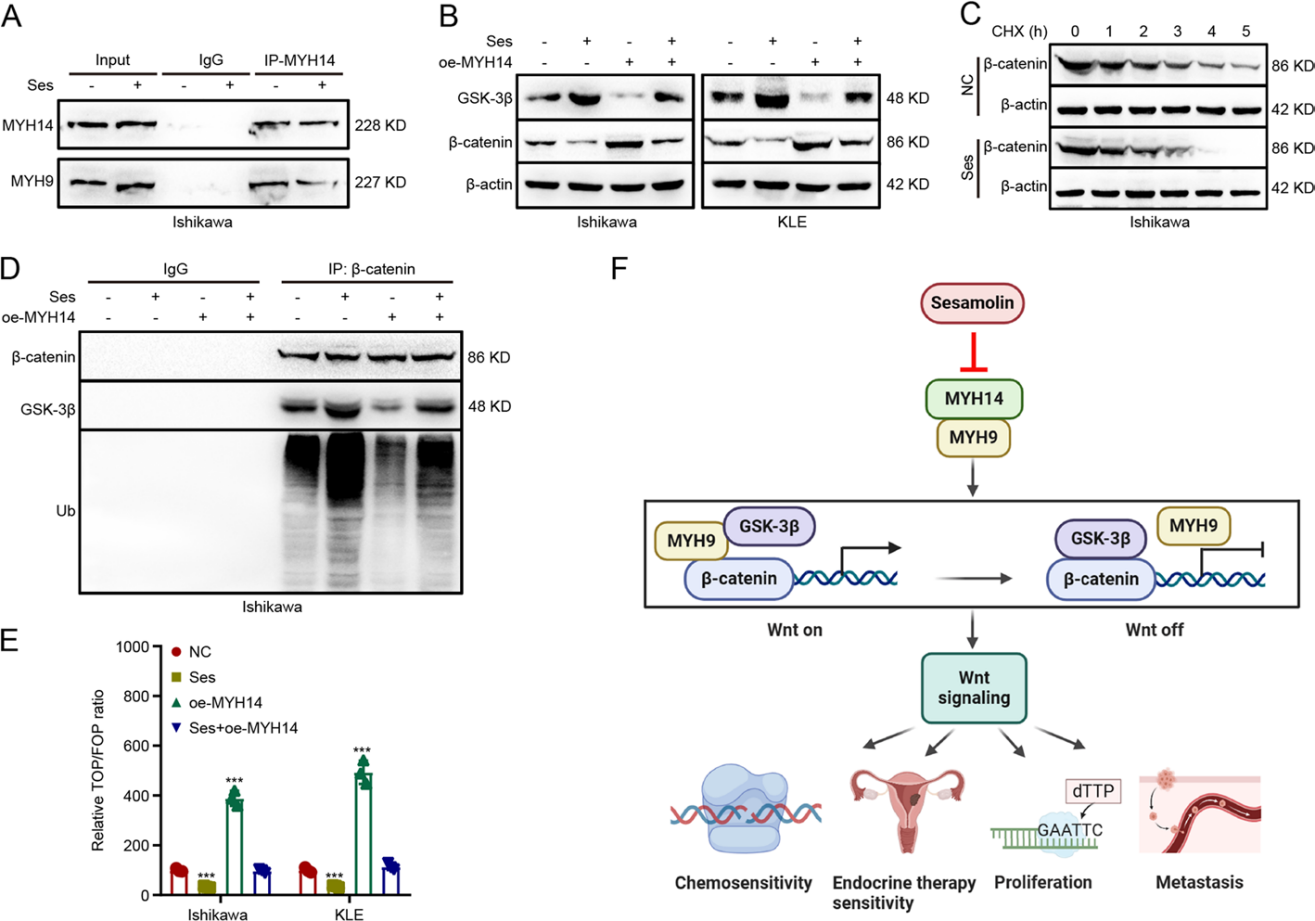
Sesamolin disrupted the interplay between MYH14 and MYH9 to inactivate Wnt/β-catenin signaling. Cells were treated with sesamolin for 48 h. **A** Co-IP assays were performed for identifying the effect of sesamolin on the interaction between MYH14 and MYH9. **B** Western blot assays were conducted for showing the effect of sesamolin and MYH14 on GSK3β and β-catenin expression. **C** CHX chase assay was adopted to present the effect of sesamolin on the half-life of β-catenin protein. **D** Co-IP assays were used for revealing the effect of sesamolin and MYH14 on the formation of GSK3β/β-catenin/ubiquitin complex in Ishikawa cells treated with MG132. **E** TOP/FOP luciferase reporter assays were adopted to assess the effect of sesamolin and MYH14 on Wnt signaling activity. **F** Working model elucidating the effect of sesamolin-targeted MYH14 and MYH9-mediated Wnt/β-catenin signaling on chemosensitivity, endocrine therapy sensitivity, proliferation, and metastasis. This graph was generated using the Biorender website (https://biorender.com/). *** *P* < 0.001 versus the control group. *CHX* cycloheximide, *Co-IP* co-immunoprecipitation, *Ses* sesamolin

Because sesamolin could mimic the inhibitory effect of MYH14 knockdown in EC progression, the effect of sesamolin on MYH14-mediated downstream signaling was subsequently determined to elucidate the mechanism of sesamolin in controlling EC progression. Co-IP assays reported that sesamolin could interfere with the interaction between MYH14 and MYH9 (Fig. 8A). Furthermore, sesamolin treatment upregulated GSK3β expression and downregulated β-catenin expression, which could be rescued by MYH14 overexpression (Fig. 8B). CHX chase assay showed that the half-life of β-catenin protein decreased due to sesamolin stimulation (Fig. 8C). Furthermore, co-IP showed that sesamolin treatment increased interaction between GSK3β, ubiquitin, and β-catenin, suggesting the formation of GSK3β/β-catenin/ubiquitin complex. TOP/FOP luciferase reporter indicated that the inactivation of Wnt signaling could be facilitated by sesamolin treatment. Nevertheless, the effect of sesamolin on MYH14-mediated downstream signaling could be reversed by MYH14 overexpression (Fig. 8D, E).

Altogether, these results suggested that sesamolin increased the chemosensitivity and endocrine therapy sensitivity of EC and repressed its proliferation and metastasis via the inhibition of MYH14-mediated downstream signaling.

## Discussion

EC chemoresistance contributes to poor patient prognosis [33]. Progesterone resistance limited the efficiency of conservative therapy in patients with EC, especially in those suffering from advanced and recurrent EC [34]. The enhancement of EMT is regarded as a crucial mechanism of EC chemoresistance and progesterone resistance [35, 36]. Targeting myosin II, a member of the myosin family, could reduce chemoresistance in gynecological cancer [37]. In this study, we used machine learning techniques and identified a novel prognosis-associated myosin family member in patients with EC, namely MYH14. MYH14 decreased the overall survival of patients with EC. Furthermore, MYH14 impaired the chemosensitivity and endocrine therapy sensitivity of EC, increased the proliferation and metastasis of EC, and upregulated the expression of β-catenin (an EMT marker) [38] by triggering MYH9-mediated Wnt signaling. Interestingly, sesamolin, a natural compound, could directly target MYH14 to attenuate Wnt/β-catenin signaling, thus repressing EC progression.

Previous studies have shown that the ssGSEA algorithm can be used to establish novel indexes based on specific genesets. Zheng et al. showed the ability of the ssGSEA-based stemness index to aid the appointment of tumor grade and its possible therapeutic and diagnostic applications. The relative expression ordering (REO) based stemness indexes were REO-based signatures with high robustness against the batch effects and can be stably used in independent datasets [39]. Yi et al. reported that computationally derived Ras-dependency indexes (RDI) can represent a measure of Ras dependency in both patient samples and cancer cell lines. Furthermore, the ssGSEA score correlated with the original RDI datasets [40]. Therefore, we calculated the myosin activity on the basis of the geneset containing 52 myosin family members and counted the chemoresistance index as per the platinum drug resistance geneset extracted from KEGG. The negative correlation between calculated myosin activity and the prognosis of patients with EC suggested the participation of myosins in EC progression. Using machine learning techniques along with WGCNA, we identified MYH14 as the most significant myosin correlating with the prognosis of patients with EC. Furthermore, bioinformatics analyses of public data and IHC analysis of our patients with EC confirmed that MYH14 expression was upregulated in EC tissues and independently predicted the poor overall survival of patients with EC.

TGF-β signaling, Wnt/β-catenin signaling, and PI3K/AKT signaling are well-established promoters of EMT [41], with EMT being a determinant of cancer cell chemoresistance [42]. In the present study, the positive association between TGF-β signaling, Wnt/β-catenin signaling, PI3K/AKT signaling, EMT, and MYH14 expression determined by bioinformatics analyses prompted us to investigate the role of MYH14 in EC chemoresistance. The oncoPredict package in the R and GSCA database has been successfully used to estimate cancer chemoresistance [43, 44]. We also used the mentioned strategy and predicted MYH14 as a promoter of EC chemoresistance. A previous study reported the involvement of Wnt signaling and EMT in progesterone resistance [45]. Therefore, we proposed the potential effect of MYH14 on EC sensitivity to progesterone. EMT is also implicated in the changing process of cell proliferation and metastasis [46, 47]. In our subsequent experiments, we confirmed that MYH14 decreased EC chemosensitivity and endocrine therapy sensitivity while promoting EC proliferation and metastasis. The expression of β-catenin is regarded as an EMT marker [38]. Thus, the stimulative role of MYH14 on β-catenin expression further confirmed MYH14 as an EMT promoter. Our previous studies reported the key effect of the Wnt/β-catenin signaling pathway on cancer chemoresistance, proliferation, and metastasis. Additionally, MYH9 was identified as an activator of Wnt signaling by inhibiting the GSK-3β-mediated ubiquitination and degradation of β-catenin [9, 28, 48, 49]. In the present study, we found the interaction between MYH14 and MYH9, with MYH14 facilitating MYH9-regulated downstream signaling to improve EC progression. We found that MYH14 promoted Wnt/β-catenin signaling-mediated EMT, thereby reducing EC chemosensitivity and endocrine therapy sensitivity while increasing EC proliferation and metastasis. These results contribute to our understanding of Wnt/β-catenin signaling in cancer progression and suggest MYH14 as a potential target for EC therapy.

The absence of well-defined targets poses a challenge to the clinical translation of existing natural compounds [50]. Protein–protein interaction (PPI) plays a crucial role in various biological processes, including catalysis, transport, and signaling. In modern drug discovery, the development of inhibitors targeting PPI has emerged as a viable approach [51–53]. Sesamolin, extracted from *Sesamum indicum* (L.) [54], has been reported to exhibit anti-cancer activities against human cancers [21]. Sesamolin could inhibit cancer proliferation, migration, and invasion [55, 56]. In this study, we demonstrated that sesamolin functioned as a PPI inhibitor, inhibiting the interaction between MYH14 and MYH9 by directly targeting MHY14. Sesamolin also facilitated the GSK-3β-mediated ubiquitination and degradation of β-catenin, leading to the inactivation of Wnt/β-catenin signaling. This inhibitory effect significantly affects the malignant phenotype of EC cells. In line with previous reports [57, 58], we identify MYH14 as a specific therapeutic target, further emphasizing sesamolin as a promising drug for EC therapy. A previous study reported that sesamolin could increase plasma γ-tocopherol and inhibit vitamin E degradation in humans without side effects in clinical studies [21]. Our study further supported that sesamolin could be applied as a potential compound for EC therapy without obvious side effects. As a major lignan in sesame seed oil, sesamolin holds significant economic and nutritional importance worldwide [59]. Sesamolin is easily available and can be used as a lead compound for the development of new drugs [60]. The existing evidence indicates sesamolin as a novel and promising therapeutic drug for the treatment of EC.

## Conclusions

In this study, we showed that MYH14 was a novel oncogene associated with EC prognosis and therapy sensitivity, independently contributing to poor overall survival of patients with EC. MYH14 interacted with MYH9, inhibiting the GSK-3β-mediated ubiquitination and degradation of β-catenin. This interaction activated the Wnt/β-catenin signaling pathway, thereby promoting EC progression. Furthermore, MYH14 could be directly targeted by sesamolin which disrupted MYH14-mediated downstream signals, thus improving the chemosensitivity and endocrine therapy sensitivity of EC while also suppressing the proliferation and metastasis of EC (Fig. 8F). Effective chemotherapy sensitization can inhibit the escape of tumor cells and mitigate serious side effects. Co-administration of sesamolin or a MYH14 inhibitor and carboplatin or paclitaxel presents a promising synergistic strategy for the treatment of advanced and recurrent EC. To conclude, our study reports MYH14 as a potential therapeutic target and sesamolin as a valuable natural compound for EC therapy.

### Supplementary Information

**Additional file 1: Fig. S1.** WGCNA revealed the prominent genes correlated with EC according to the GSE17025 dataset. **Fig. S2.** The association between the biological processes, signaling pathways, and MYH14 expression in EC. **Fig. S3.** MYH14 levels were potentially associated with EC chemoresistance. **Fig. S4.** Effects of carboplatin and paclitaxel on EC cell viability. **Table S1.** A list of antibodies used in this study. **Table S2.** The primers used in this study. **Table S3.** A list of genes belonging to the myosin family. **Table S4.** The correlation between MYH14 and Ki67 expression in endometrial cancer.

## Data Availability

The datasets used and/or analyzed during the current study are available from the corresponding author on reasonable request.

## Abbreviations

CBP: Carboplatin
CCK-8: Cell counting kit 8
CETSA: Cellular thermal shift assay
CHX: Cycloheximide
Co-IP: Co-immunoprecipitation
EC: Endometrial cancer
EMT: Epithelial-mesenchymal transition
FBS: Fetal bovine serum
GEO: Gene Expression Omnibus
GSCA: Gene Set Cancer Analysis
IHC: Immunohistochemistry
KEGG: Kyoto Encyclopedia of Genes and Genomes
MPA: Medroxyprogesterone acetate
PPI: Protein–protein interaction
PTX: Paclitaxel
qRT-PCR: Quantitative reverse transcription polymerase chain reaction
Ses: Sesamolin
ssGSEA: Simple sample gene set enrichment analysis
TCGA: The Cancer Genome Atlas
UCEC: Uterine corpus endometrioid carcinoma
WGCNA: Weighted gene co-expression network analysis
