# Supplementary material for: Sesamolin serves as an MYH14 inhibitor to sensitize endometrial cancer to chemotherapy and endocrine therapy via suppressing MYH9/GSK3β/β-catenin signaling
Source: Cell Mol Biol Lett. 2024 May 2;29:63. doi: 10.1186/s11658-024-00583-9 (PMC11067147; doi:10.1186/s11658-024-00583-9)
Supplement: Supplementary file 1 — Additional file 1: Fig. S1. WGCNA revealed the prominent genes correlated with EC according to the GSE17025 dataset. Fig. S2. The association between the biological processes, signaling pathways, and MYH14 expression in EC. Fig. S3. MYH14 levels were potentially associated with EC chemoresistance. Fig. S4. Effects of carboplatin and paclitaxel on EC cell viability. Table S1. A list of antibodies used in this study. Table S2. The primers used in this study. Table S3. A list of genes belonging to the myosin family. Table S4. The correlation between MYH14 and Ki67 expression in endometrial cancer. [file 11658_2024_583_MOESM1_ESM.doc]

**Additional Material for**

**Sesamolin serves as an MYH14 inhibitor to alleviate therapeutic resistance of endometrial cancer via suppressing MYH9/GSK3β/β-catenin signaling**

Yibin Lin ^1, #^, Xiao Chen ^2, 3, #^, Linping Lin ^4^, Benhua Xu ^5, *^, Xiaofeng Zhu ^6, *^, Xian Lin ^7, 8, *^

^1^ Department of Gynecology, Clinical Oncology School of Fujian Medical University, Fujian Cancer Hospital, Fuzhou 350014, Fujian, China

^2^ Department of Intensive Care Unit, First Affiliated Hospital of Fujian Medical University, Fuzhou 350001, Fujian, China

^3^ Department of Intensive Care Unit, National Regional Medical Center, Binhai Campus of the First Affiliated Hospital, Fujian Medical University, Fuzhou 350001, Fujian, China

^4^ Hunan Institute of Engineering, Xiangtan [411100](https://ditu.so.com/zt/postcode.html?cityid=430300&src=onebox-map_new_youbian-postcoad), Hunan, China

^5^ Department of Radiation Oncology, Fujian Medical University Union Hospital, Fuzhou 350001, Fujian, China

^6^ Department of Oral Maxillo-Facial Surgery, The First Affiliated Hospital of Fujian Medical University, Fuzhou 350001, Fujian, China

^7^ Shenzhen Key Laboratory of Inflammatory and Immunology Diseases, Shenzhen 518036, Guangdong, China

^8^ Peking University Shenzhen Hospital, Shenzhen 518036, Guangdong, China

^#^ These authors have contributed equally to this work

* Correspondence:

**Xian Lin**, Shenzhen Key Laboratory of Immunity and Inflammatory Diseases, Peking University Shenzhen Hospital, 1120 Lianhua Road, Futian District, Shenzhen 518036, Guangdong, China. Telephone: +86-15626040245. Fax: +86-0755-83061340. Email: linxiangabriel@fjmu.edu.cn

This file includes:

**Additional file 1: Fig. S1-4 and Additional file 1: Table S1-****4.**


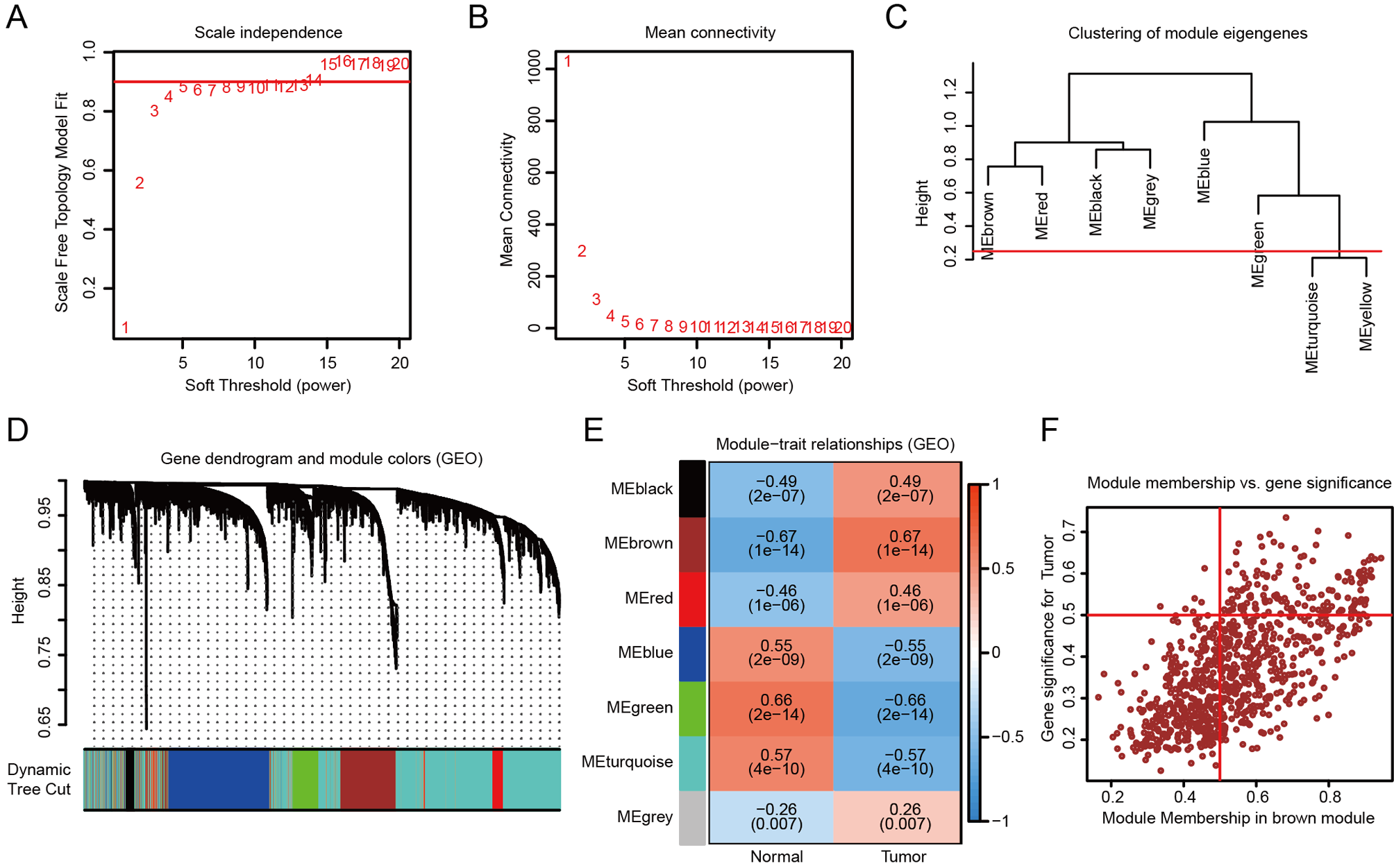


**Fig. S1** WGCNA revealed the prominent genes correlated with EC according to the GSE17025 dataset. (**A, B**) The scale independence and mean connectivity were plotted to reveal the soft threshold and scale-free topology model fit index as per GSE17025 dataset. (**C, D**) A GeneTree and a cluster dendrogram were established based on the soft threshold according to the GSE17025 dataset. Different colors represent different co-expression modules. (**E**) The heatmap showing the association between gene modules and EC according to the GSE17025 dataset. Each row represents a module, and each column represents a clinical status. (**F**) The scatter plots presenting the relationship between genes and EC in the MEbrown module. EC: Endometrial cancer; GEO: Gene Expression Omnibus; WGCNA: Weighted gene co-expression network analysis.


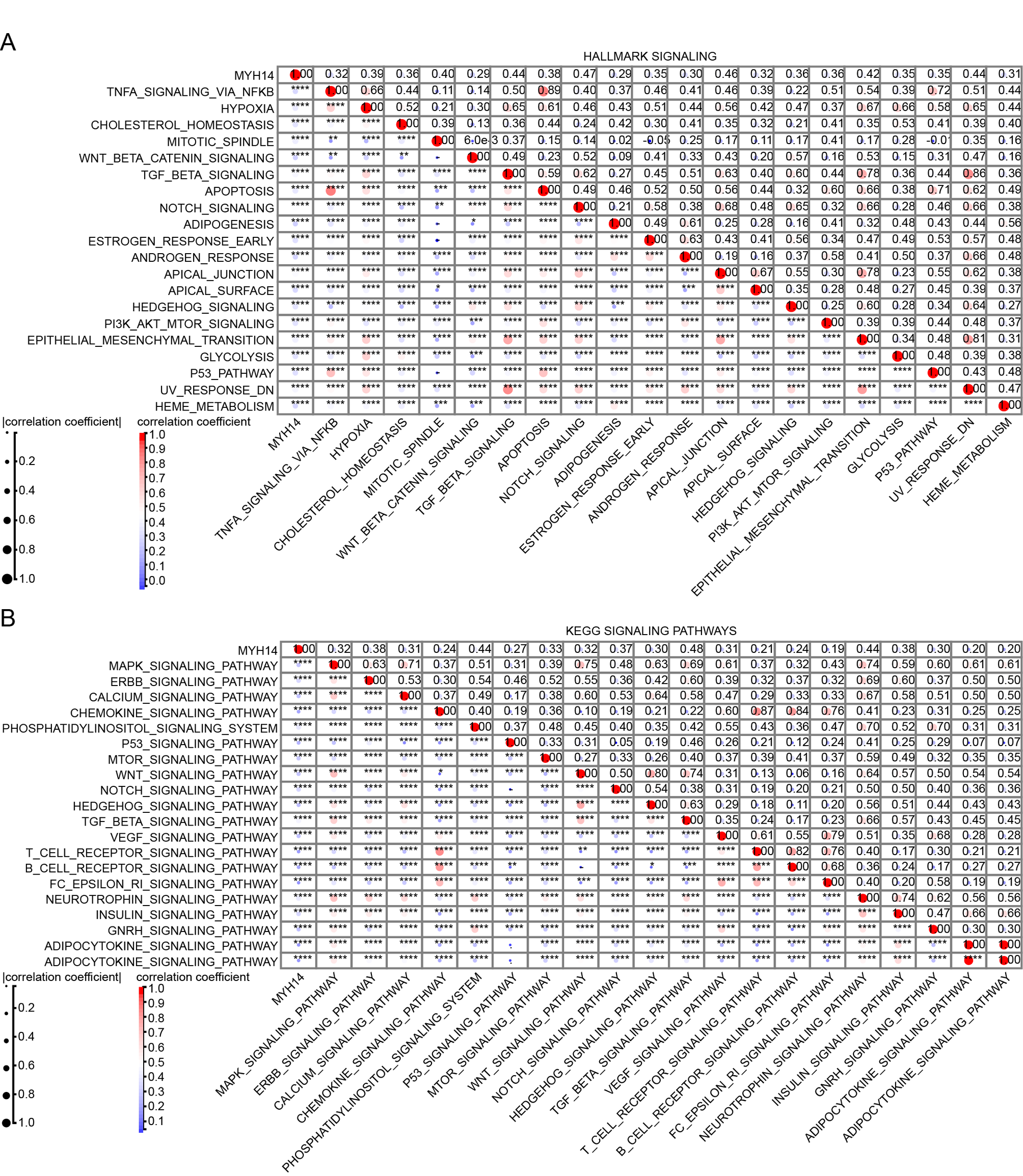


**Fig. S2** The association between the biological processes, signaling pathways, and MYH14 expression in EC. ssGSEA was conducted for calculating the activities of biological processes and signaling pathways of TCGA samples based on HALLMARK and KEGG genesets. (**A**) The correlation analyses were adopted to detect the association between TGF-β signaling, Wnt/β-catenin signaling, EMT, PI3K/AKT/mTOR signaling, Notch signaling, and MYH14 expression based on HALLMARK geneset. (**B**) The correlation analyses were applied to measure the association between TGF-β signaling, Wnt signaling, phosphatidylinositol signaling system, mTOR signaling, Notch signaling, and MYH14 expression based on KEGG dataset. EC: Endometrial cancer; EMT: Epithelial-mesenchymal transition; ssGSEA: simple sample gene set enrichment analysis; TCGA: The Cancer Genome Atlas.


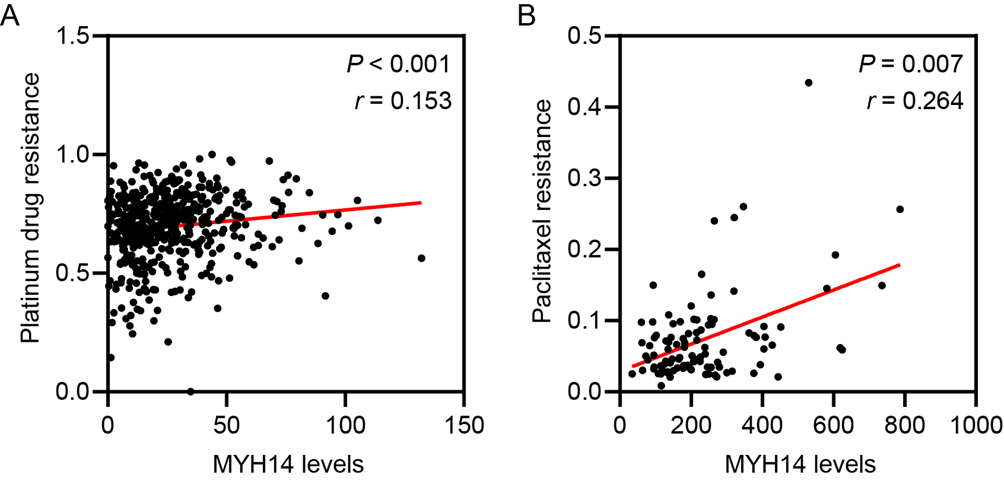


**Fig. S3** MYH14 levels were potentially associated with EC chemoresistance. (**A**) The correlation analysis was adopted for assessing the association between MYH14 expression and platinum drug resistance index in the TCGA UCEC dataset. ssGSEA was applied to calculate the platinum drug resistance index of EC patients. (**B**) The correlation analysis was used for measuring the association between MYH14 expression and paclitaxel drug resistance in GSE17025 dataset. OncoPredict in R was used to calculate paclitaxel drug resistance and reveal the correlation between MYH14 expression and paclitaxel drug resistance. EC: Endometrial cancer; ssGSEA: simple sample gene set enrichment analysis; TCGA: The Cancer Genome Atlas; UCEC: Uterine corpus endometrioid carcinoma.


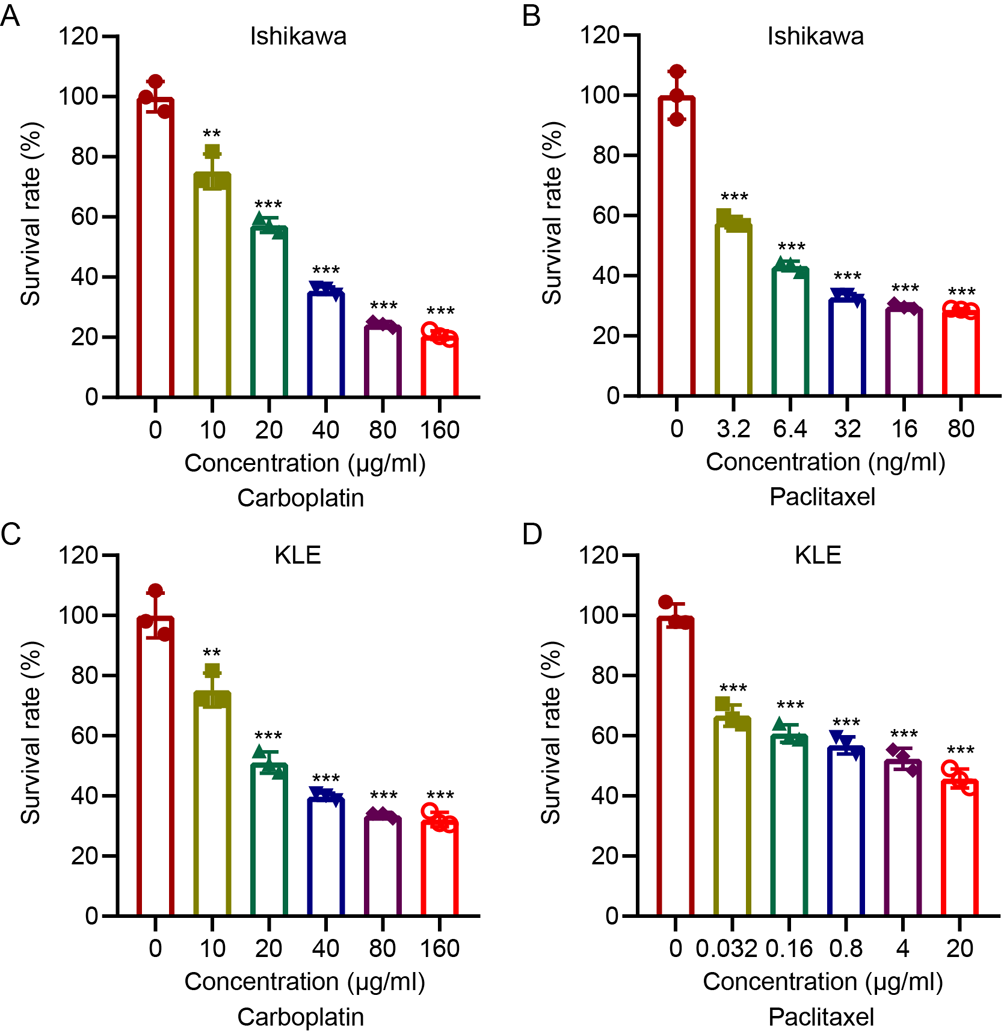


**Fig. S4** Effects of carboplatin and paclitaxel on EC cell viability. Cells were treated with carboplatin or paclitaxel for 48 h. (**A-D**) CCK-8 assays were conducted for assessing cell viability in carboplatin-treated Ishikawa and KLE cells, paclitaxel-treated Ishikawa and KLE cells, and the controls. ***P* < 0.01, ****P* < 0.001 vs the control group. EC: Endometrial cancer.

| **Table S1** A list of antibodies used in this study | | | | |
| --- | --- | --- | --- | --- |
| Antibodies | Cat. No | Company | Species | Application |
| MYH14 | 20716-1-AP | Proteintech | Rabbit | IHC, WB, Co-IP |
| MYH9 | 11128-1-AP | Proteintech | Rabbit | WB |
| GSK-3β | 22104-1-AP | Proteintech | Rabbi | WB |
| β-catenin | 51067-2-AP | Proteintech | Rabbit | WB, Co-IP |
| ubiquitin | Proteintech | Proteintech | Rabbit | WB |
| β-actin | 4970T | CST | Rabbit | WB |
| Phospho-Histone H2A.X (Ser139) | 9718S | CST | Rabbit | IF |

IHC: Immunohistochemistry, WB: Western blot, Co-IP: Co-Immunoprecipitation, IF: Immunofluorescence

| **Table S2** The primers used in this study | | |
| --- | --- | --- |
| Primers |  | Sequence (5’-3’ ) |
| MYH14 | Forward | CGGCAGCGATACGAGAT |
|  | Reverse | CTGGAAGGAGACGATGATGT |
| MYH9 | Forward | GGAGCCGTACAACAAATACC |
|  | Reverse | AGAACCCCTGAGATGACCC |
| β-actin | Forward | CTCGCTGTCCACCTTCCA |
|  | Reverse | ACCTTCACCGTTCCAGTTTT |

| **Table S3** A list of genes belonging to the myosin family | | | | | |
| --- | --- | --- | --- | --- | --- |
| Myosin family members | | | | | |
| MYH7 | MYO6 | MYO1C | MYO1B | MYLPF | MYO3B |
| MYH9 | MYH14 | MYO9B | MYH15 | MYO18A | MYL10 |
| MYH6 | MYH8 | MYH4 | MYO5B | MYO5C |  |
| MYH2 | MYL9 | MYL1 | MYL7 | MYO1H |  |
| MYO5A | MYO1A | MYO15A | MYO3A | MYO7B |  |
| MYH10 | MYL4 | MYL6 | MYO1F | MYO1D |  |
| MYL2 | MYO10 | MYO1E | MYL6B | MYO16 |  |
| MYO7A | MYL3 | MYO9A | MYL5 | MYO18B |  |
| MYH11 | MYH1 | MYL12B | MYL12A | MYO15B |  |
| MYH3 | MYH13 | MYH7B | MYO1G | MYO19 |  |

| **Table S4** The correlation between MYH14 and Ki67 expression in endometrial cancer | | | | | |
| --- | --- | --- | --- | --- | --- |
| **MYH14 expression** | **Ki67 expression** | | **Total** | ***Kappa*** | ***P* value** |
|  | **Negative** | **Positive** |  |  |  |
| **Low** | 13 (34.2%) | 25 (65.8%) | 38 | 0.206 | 0.032 |
| **High** | 7 (14.6%) | 41 (85.4%) | 48 |
| **Total** | 20 | 66 | 86 |  |  |
